# Insertion of a G-quadruplex or hairpin structure into the 5' UTR or poly(A) sequences reduces translation efficiency of the encephalomyocarditis virus internal ribosome entry site: a preclinical *in vitro* study

**DOI:** 10.12771/emj.2025.00423

**Published:** 2025-05-27

**Authors:** Yun Ji Kim, So-Hee Hong

**Affiliations:** Department of Microbiology, Ewha Womans University College of Medicine, Seoul, Korea

**Keywords:** Encephalomyocarditis virus, Internal ribosome entry sites, Messenger RNA, Transfection

## Abstract

**Purpose:**

Internal ribosome entry site (IRES) elements, present in both viral and cellular messenger RNAs (mRNAs), facilitate cap-independent translation by recruiting ribosomes to internal regions of mRNA. This study aimed to investigate the impact of inserting G-quadruplex and hairpin structures into the 5' untranslated region (UTR) and poly(A) sequences on the translation efficiency of the encephalomyocarditis virus (EMCV) IRES, using an IRES-based RNA platform encoding OX40L, 4-1BBL, and GFP.

**Methods:**

G-quadruplex and hairpin structures, derived from HIV-1 (human immunodeficiency virus type 1) or custom-designed, were synthesized and inserted into the 5' UTR and poly(A) tail regions of EMCV IRES vectors. These constructs were amplified by polymerase chain reaction, ligated into plasmids, and transcribed *in vitro*. B16 melanoma, TC-1 tumor, and HEK293 cells were transfected with these RNA constructs. Protein expression levels were assessed at 6, 12, and 24 hours post-transfection by flow cytometry and fluorescence microscopy. Statistical analyses employed one-way analysis of variance with the Dunnett test.

**Results:**

The insertion of G-quadruplex and hairpin structures altered RNA secondary structure, significantly reducing protein expression. In the 5' UTR, the G-quadruplex nearly abolished OX40L expression (1.18%±0.41% at 6 hours vs. 18.23%±0.16% for control), while the hairpin structure reduced it (16.29%±1.46% vs. 22.84%±1.17%). In the poly(A) tail region, both structures decreased GFP expression across all cell lines (4.86%±1.35% to 7.27%±0.32% vs. 39.56%±2.07% in B16 cells).

**Conclusion:**

Inserting G-quadruplex and hairpin structures into EMCV IRES UTRs inhibits translation efficiency, suggesting the need for precise RNA structure modeling to enhance IRES-mediated translation.

## Introduction

### Background

Internal ribosome entry sites (IRESs) are RNA elements that recruit ribosomes to internal regions of messenger RNAs (mRNAs), enabling translation through a cap-independent pathway [1,2]. IRESs were first discovered in viruses belonging to the Picornaviridae family, such as poliovirus (PV) and encephalomyocarditis virus (EMCV), in the late 1980s [3]. Subsequently, numerous IRESs have been identified in viral and cellular mRNAs [4]. During viral infection or cellular stress, various mechanisms often suppress conventional cap-dependent translation [1]. Under these adverse conditions, viruses and certain cellular mRNAs rely on IRESs to maintain protein translation [2]. Compared with cap-dependent expression systems, viral IRES platforms offer advantages such as bicistronic gene expression, cap independence, and flexible design suitable for therapeutic applications [5].

The regulation of protein expression is a complex process involving multiple levels of control [6]. Recent studies have emphasized the crucial role of secondary RNA structural elements in fine-tuning translation efficiency [7,8]. Among these elements, G-quadruplexes and hairpin structures have gained particular attention due to their ability to improve protein production under various conditions, such as infection, hypoxia, and DNA damage [2,3].

G-quadruplexes are 4-stranded helical structures formed in guanine-rich, single-stranded RNA (ssRNA) sequences. They function similarly to IRESs and promote cap-dependent translation initiation [9-11]. The G-quadruplex follows a structural algorithm characterized by the sequence GXN1-7GXN1-7GXN1-7GXN1-7 (X ≥3, N represents any nucleotide), consisting of guanine quartets (G-quartets) surrounded by loop regions [12]. G-quadruplex structures are frequently found in proto-oncogenes related to cancer, such as c-myc, VEGF, and Bcl-2 [13]. These structures are thermodynamically stable, easily formed, and contribute to RNA stability [12,14]. Under cellular stress conditions, G-quadruplex structures help maintain RNA-ribosome interactions, thereby supporting continuous protein translation [10]. They are also well known for influencing gene splicing and enhancing the binding of specific transcription factors [10,13]. A recent study has shown that increasing the loop size of G-quadruplexes enhances protein translation [6].

The RNA hairpin structure resembles a loop or U-shape and forms when 2 regions of the same RNA strand base-pair to create a double helix [15]. Under specific conditions, such as hypoxia and DNA damage, hairpin structures can enhance start codon recognition, thereby increasing protein expression [7,16]. Hairpin structures also contribute to RNA stability by forming stable secondary structures at mRNA termini, preventing degradation, and extending RNA half-life, which ultimately enhances overall protein expression [17,18]. Notably, the use of multiple hairpin structures rather than a single structure yields superior translational outcomes [17].

Additionally, other studies have demonstrated that the combined use of hairpins and G-quadruplex structures significantly enhances translation efficiency [6,17].

### Objectives

Based on this evidence, in the present study, we designed and inserted G-quadruplexes and hairpin structures into the EMCV IRES and assessed changes in the protein expression levels of the IRES-encoded gene to reveal the effects of these inserted structures on the translational function of the IRES.

## Methods

### Ethics statement

This study constitutes laboratory research, not involving human subjects. Therefore, neither institutional review board approval nor informed consent was required.

### Design and synthesis of G-quadruplex and hairpin structures

Plasmids encoding 4-1BBL, OX40L, and GFP, based on the EMCV IRES, were used as the backbone, and these vectors were constructed according to previously described cloning methods [5]. The G-quadruplex and hairpin sequences were designed based on human immunodeficiency virus type 1 (HIV-1)-derived sequences, with fundamental structural details and custom-designed sequences described in Table 1.

All genes for structural design were synthesized by Cosmo Genetech Inc. (https://www.cosmogenetech.com). The inserted G-quadruplex and hairpin genes were amplified by polymerase chain reaction (PCR) using the following reaction conditions: 10 ng of plasmid template, 0.5 μL of 10 pmol forward primer, 0.5 μL of 10 pmol reverse primer, 4 μL of Phusion 5× buffer, 0.4 μL of dNTPs, and 0.2 μL of Phusion enzyme (Thermo Fisher Scientific; https://www.thermofisher.com).

The following primers were used for PCR:

E-H forward: 5′- CCGAATTCTAATACGACTCACTAT -3′

E-H reverse: 5′- CCCCTAGGAATGCTCGTCAAG -3′

E-G forward: 5’- CCGAATTCTAATACGACTCACTAT -3′

E-G reverse: 5’- CCCCTAGGAATGCTCGTCAAG -3’

E-M1 forward: 5′- CCGTCGACCGATCGTAGTGTAGTCAC -3′

E-M1 reverse: 5′- CCGCGGCCGCGCTAGC -3′

E-M2 forward: 5′- CCGTCGACCGATCGTAGTGTAGTCAC -3′

E-M2 reverse: 5′- CCGCGGCCGCTGGTAATG -3′

E-M3 forward: 5′- CCGTCGACCGATCGTAGTGTAGTCAC -3′

E-M3 reverse: 5′- CCGCGGCCGCCAGGCT -3′

E-M4 forward: 5’- CCGTCGACCGATCGTAGTGTAGTCAC -3’

E-M4 reverse: 5’- CCGCGGCCGCCAGGCT -3’

The PCR conditions were as follows: initial denaturation at 98°C for 30 seconds, followed by 35 cycles of denaturation at 98°C for 10 seconds, annealing at 55°C for 10 seconds, and extension at 72°C for 20 seconds. A final extension was performed at 72°C for 1 minute, followed by indefinite storage at 12°C. Amplified products were analyzed using 2% agarose gel electrophoresis, and product sizes were verified using a 50 bp DNA ladder (Dyne LoadingSTAR+50 bp DNA Ladder; Dynebio; http://www.dynebio.co.kr). Target bands were excised and purified using a gel cleanup kit. The purified gene fragments were digested with respective restriction enzymes (Enzynomics; https://www.enzynomics.com) at 37°C for 1 hour and 30 minutes, followed by enzyme inactivation at 65°C for 30 minutes. After additional purification, the amplified genes were ligated into the pALpA_EMCV IRES vector using T4 ligase (RBC Rapid Ligation Kit, RBC, Taiwan) and incubated at 4°C overnight. The ligation products were transformed into Escherichia coli DH5α (Enzynomics) by heat shock at 42°C. Ampicillin-resistant colonies were selected and cultured in LB medium (Duchefa; https://www.duchefa-farma.com) containing ampicillin (Duchefa). Finally, plasmid DNA was purified using a Plasmid DNA Miniprep S&V kit (Bionics; https://www.bionicsro.co.kr). The final plasmids were confirmed by electrophoresis following digestion with restriction enzymes.

### *In vitro* transcription

DNA templates were linearized using the NotI restriction enzyme (Enzynomics). *In vitro* transcription was performed using the EZ(TM) T7 High Yield In-Vitro Transcription Kit (Enzynomics), driven by the T7 promoter. A 1 μg linearized DNA template was mixed with T7 transcription buffer, MgCl_2_, 10 mM dithiothreitol, enhancer solution, 5 mM ribonucleoside triphosphates, 200 U of T7 polymerase mix, and ultrapure water, reaching a final reaction volume of 20–100 μL. The reaction mixture was incubated at 37°C for 4–6 hours.

Following transcription, DNA was removed by DNase I (Promega; https://promega.com) treatment at 37°C for 30 minutes. RNA was precipitated using lithium chloride, and double-stranded RNA was eliminated via cellulose purification following previously established methods [19]. RNA purity and concentration were assessed using a NEO-Nabi UV-VIS Nano spectrophotometer (MicroDigital Co. Ltd.; https://www.md-best.com). Only samples with 260/230 and 260/280 absorbance ratios >1.9 were used for subsequent analyses. RNA was mixed with denaturing dye, heated at 70°C for 10 minutes, and analyzed by electrophoresis on a 1.5% agarose gel. RNA was stained with RedSafe Nucleic Acid Staining Solution, and quality was assessed using the RiboRuler High Range RNA Ladder (Thermo Fisher Scientific).

### Cell culture

Mouse B16 melanoma (CRL-6475; ATCC; https://www.atcc.org) and HEK293 cells (Korean Cell Line Bank; https://cellbank.snu.ac.kr) were cultured in Dulbecco’s modified Eagle’s medium (DMEM; GenDEPOT; https://gendepot.com) supplemented with 10% fetal bovine serum (FBS; Welgene; https://www.welgene.com) and 1% penicillin/streptomycin (Welgene).

Mouse TC-1 tumor cells (CRL-2493; ATCC) were maintained in RPMI 1640 medium (Welgene) supplemented with 10% FBS and 1% penicillin/streptomycin. All cells were incubated at 37°C in a humidified atmosphere containing 5% CO_2_.

### Transfection

B16, TC-1, and HEK293 cells (7×10^5^ cells/well) were seeded into 6-well plates (SPL; http://www.spllifesciences.com) and cultured in DMEM or RPMI 1640 medium (supplemented with 10% FBS and 1% penicillin/streptomycin) at 37°C with 5% CO_2_ for 12 hours. After incubation, cells were washed twice with cold phosphate-buffered saline (PBS) and transfected with 5 μg of RNA using Lipofectamine 3000 (Thermo Fisher Scientific) in Opti-MEM (Gibco, Thermo Fisher Scientific) and serum-free medium. Protein expression was assessed by flow cytometry and live imaging using a Leica Thunder Imager (Leica; https://www.leica-microsystems.com).

### Flow cytometry analysis

Cells were harvested and resuspended in flow cytometry buffer (PBS containing 1% BSA and 0.01% NaN_3_). Fc receptors were blocked by incubation with anti-mouse CD16/32 (TruStain FcX, BioLegend) at 4°C for 15 minutes. Subsequently, cells were stained at 4°C for 30 minutes in the dark with antibodies and dye: anti-mouse CD275 (ICOS Ligand, clone HK5.3, BioLegend), anti-4-1BBL (CD137L, clone TKS-1, BioLegend), anti-CD252 (OX40L, clone RM143L, BioLegend), and LIVE/DEAD Fixable Aqua Dead Cell Stain (Invitrogen).

### Statistical methods

Statistical significance was evaluated using one-way analysis of variance followed by the Dunnett post hoc multiple comparisons test. A P-value less than 0.05 was considered statistically significant (P<0.05, P<0.01, P<0.001). Data are expressed as mean±standard deviation (SD). Analyses were conducted using GraphPad Prism ver. 10.0 (GraphPad Software).

## Results

### Design and insertion of G-quadruplex and hairpin structures in IRES-based vectors: impact on IRES RNA secondary structure

**Fig. 1.**
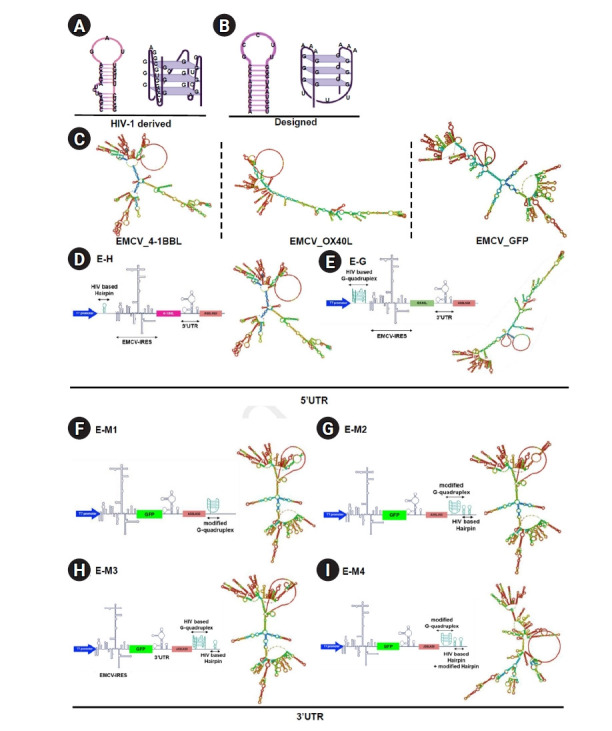
Design and positioning of secondary RNA structures inserted into the encephalomyocarditis virus (EMCV) internal ribosome entry site (IRES) and their resulting secondary structures. (A) Structure of human immunodeficiency virus type 1 (HIV-1) derived hairpin and G-quadruplex. (B) Structure of designed hairpin and G-quadruplex. (C) Predicted structure of the EMCV IRES-based RNA platform encoding 4-1BBL, OX40L, or GFP using RNAfold program. (D) Design and predicted 2-dimensional (2D) structure of the EMCV IRES-based RNA platform expressing 4-1BBL and containing a hairpin structure in the 5’ untranslated region (UTR). (E) Design and predicted 2D structure of the EMCV IRES-based RNA platform expressing OX40L and containing a G-quadruplex structure in the 5’ UTR. (F–I) Design and predicted 2D structure of the EMCV IRES-based RNA platform expressing GFP and containing a secondary structure in the poly(A) tail.

In this study, 2 different G-quadruplex and hairpin structures were employed: one derived from an HIV-1 sequence and another custom-designed specifically for this study (Fig. 1A, B). To investigate the effects of inserting these structures into the untranslated regions (UTRs) of an IRES-based platform, they were integrated into an EMCV IRES-based RNA vector (Fig. 1C). First, RNA secondary structures derived from HIV-1, previously described in earlier studies [13,20], were synthesized and inserted into the 5' UTR of the EMCV IRES, encoding the co-stimulatory molecules OX40L and 4-1BBL. These secondary structures were positioned between the T7 promoter and the IRES sequence (Fig. 1D, E). The structural elements were directly linked to the T7 promoter and IRES without a spacer or linker. Structural prediction analyses indicated that inserting the hairpin motif minimally impacted the native IRES structure, whereas inserting the G-quadruplex significantly altered the RNA secondary structure (Fig. 1C–E).

To explore the impact of structures located in the 3' UTR on protein expression, G-quadruplex and hairpin motifs were also inserted into the 3' UTR of a GFP-encoding EMCV IRES platform (Fig. 1F–I). The secondary structures were placed downstream of the poly(A) tail, consisting of 100 adenine residues, followed by an additional 10-nucleotide poly(A) sequence. A 12-nucleotide linker sequence was also incorporated to ensure proper spatial positioning of the secondary structures. Hairpin and G-quadruplex structures with distinct sequences were designed and inserted into the same location within the 3' UTR (Fig. 1F–I). Despite sequence differences between the 2 hairpin and 2 G-quadruplex structures, structural prediction analyses demonstrated that all inserted RNA secondary constructs significantly altered the RNA conformation compared to the original vector (Fig. 1C, 1F–I).

### Reduced protein expression in EMCV IRES-based RNA platforms facilitated by G-quadruplex and hairpin insertion

**Fig. 2.**
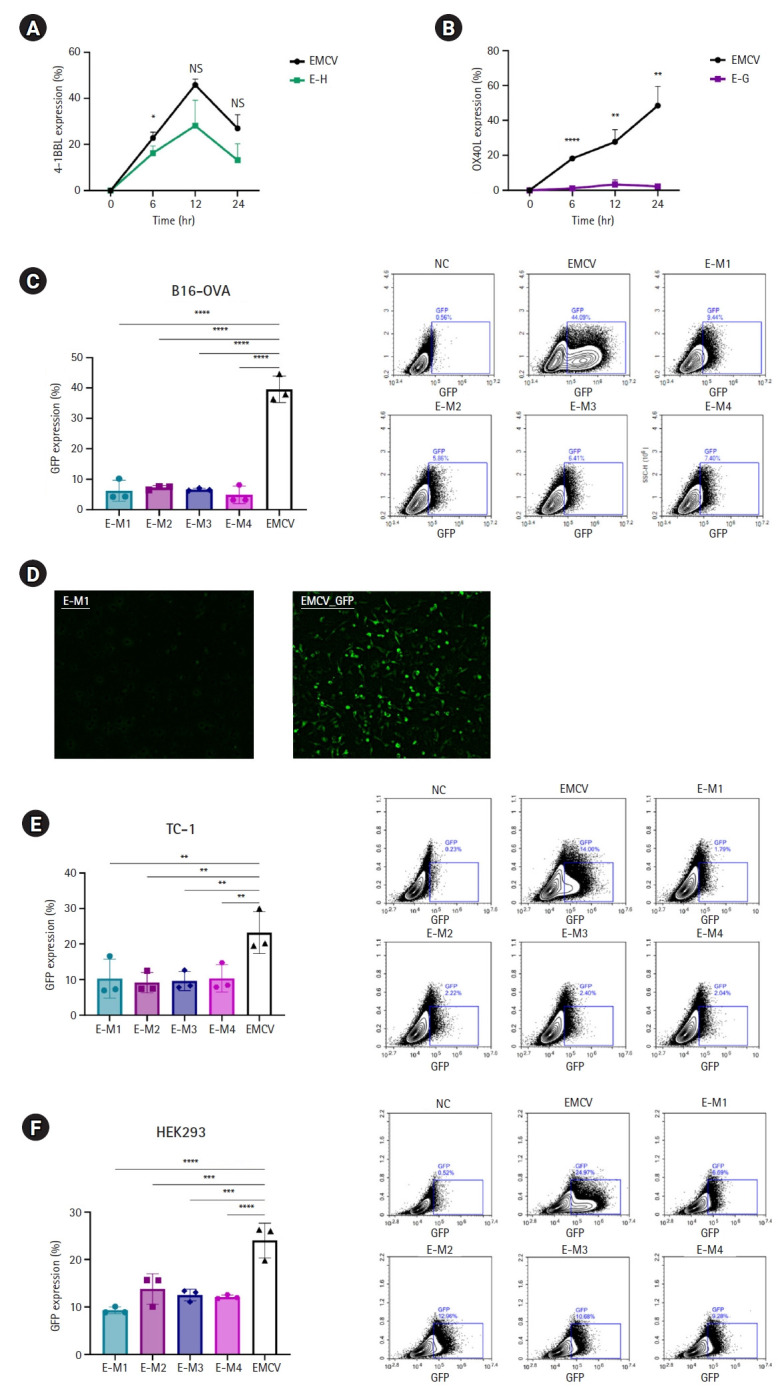
Reduced protein expression efficiency of encephalomyocarditis virus (EMCV)-internal ribosome entry site (IRES) platforms containing hairpin or G-quadruplex secondary structures. (A) Time-dependent expression levels of 4-1BBL in B16 melanoma cells transfected with EMCV-IRES-4-1BB with or without a hairpin in the 5’ untranslated region (UTR). (B) Time-dependent expression levels of OX40L in B16 melanoma cells transfected with EMCV-IRES-OX40L with or without a G-quadruplex structure in the 5’ UTR. (C) Results of flow cytometry 24 hours after transfection of the B16 melanoma cell line with EMCV-IRES-GFP, with or without structural elements in the poly(A) tail. (D) Fluorescence live imaging 24 hours after transfection of the B16 melanoma cell line with EMCV-IRES-GFP, with or without structural elements in the poly(A) tail. (E) Results of flow cytometry 24 hours after transfection of the TC-1 cell line with EMCV-IRES-GFP, with or without structural elements in the poly(A) tail. (F) Results of flow cytometry 24 hours after transfection of the HEK 293 cell line with EMCV-IRES-GFP, with or without structural elements in the poly(A) tail. NS, not significant.

To evaluate the impact of G-quadruplex and hairpin structures on translation efficiency, EMCV-IRES platforms containing these secondary structures inserted into either the 5' UTR or poly(A) tail region were transfected into various cell lines. The expression levels of encoded genes such as OX40L, 4-1BBL, or GFP were then evaluated. First, to assess the effect of structures located in the 5' UTR, EMCV-IRES encoding OX40L and 4-1BBL with inserted hairpin or G-quadruplex motifs at the 5' UTR terminal were synthesized. These constructs were transfected into B16 melanoma cells, and protein expression levels were measured at 6, 12, and 24 hours post-transfection. Compared to cells transfected with the EMCV-IRES construct lacking these secondary structures, significantly reduced protein expression levels were observed (Fig. 2A, B). Specifically, EMCV-IRES constructs containing hairpin structures resulted in decreased protein expression. At 6 hours post-transfection, EMCV control exhibited a protein expression rate (mean±SD) of 22.84%±1.17%, whereas the E-H construct displayed a reduced rate of 16.29%±1.46%. At 12 hours, the expression levels were 45.8%±1.21% (EMCV) and 28.13%±5.2% (E-H), and at 24 hours, they were 26.98%±2.77% and 13.17%±3.34%, respectively (Fig. 2A). Notably, insertion of the HIV-1 G-quadruplex structure at the 5' UTR terminal nearly abolished OX40L expression (Fig. 2B). At 6 hours post-transfection, EMCV exhibited a protein expression rate of 18.23%±0.16%, compared with only 1.18%±0.41% for the E-G construct. At 12 hours, the expression levels were 27.67%±3.33% and 3.34%±1.28%, and at 24 hours, they were 48.5%±5.19% and 2.23%±0.47%, respectively (Fig. 2B).

To determine whether these secondary structures affect protein expression regardless of their location within the UTRs, hairpin and G-quadruplex motifs were inserted downstream of the poly(A) tail in the 3' UTR of an EMCV IRES-based vector encoding GFP. These EMCV-IRES constructs were transfected into B16 melanoma, TC-1 tumor, and HEK293 human embryonic kidney cells, and GFP expression was analyzed at 24 hours post-transfection. Consistent with results observed in the 5' UTR experiments, all ssRNA constructs containing secondary structures at the poly(A) tail terminal of the 3' UTR exhibited significantly reduced GFP expression compared to the control EMCV IRES (Fig. 2C–F). In B16 cells, the EMCV IRES control exhibited a protein expression rate (mean±SD) of 39.56%±2.07%, whereas E-M1, E-M2, E-M3, and E-M4 showed notably lower expression levels: 6.23%±1.61%, 7.27%±0.32%, 6.6%±0.19%, and 4.86%±1.35%, respectively (Fig. 2C). Fluorescence microscopy confirmed these findings, showing clearly visible GFP fluorescence only in the EMCV IRES control group, whereas the E-M constructs exhibited minimal fluorescence, indicating extremely low protein expression (Fig. 2D). Similar results were obtained across other cell lines. In TC-1 cells, the EMCV IRES control showed an expression rate of 23.22%±2.76%, whereas the E-M1, E-M2, E-M3, and E-M4 constructs exhibited significantly lower expression levels: 10.28%±2.59%, 9.13%±1.37%, 9.57%±1.26%, and 10.35%±1.82%, respectively (Fig. 2E). Likewise, in HEK293 cells, the EMCV control exhibited protein expression of 24%±1.73%, compared with significantly reduced levels in E-M1, E-M2, E-M3, and E-M4 constructs: 9.27%±0.33%, 13.76%±1.52%, 12.5%±0.59%, and 12.09%±0.2%, respectively (Fig. 2F). These findings consistently demonstrated that G-quadruplex and hairpin insertions significantly reduce protein expression across different cell types (Fig. 2C–F).

## Discussion

### Key results

This study demonstrated that inserting G-quadruplex and hairpin structures into the 5' UTR and poly(A) region of an EMCV IRES-based RNA platform significantly alters RNA secondary structure and reduces protein expression. In the 5' UTR, the insertion of the G-quadruplex nearly abolished OX40L expression, whereas insertion of the hairpin structure reduced expression to a lesser extent. In the 3' UTR, both types of structures led to decreased GFP expression across B16, TC-1, and HEK293 cell lines, with expression levels dropping to as low as 4.86% compared to control constructs. Based on these results, the study suggests that insertion of G-quadruplex and hairpin motifs reduces translational efficiency in EMCV IRES-based vectors, irrespective of whether they are positioned within the 5' or 3' UTR.

### Interpretation/comparison with previous studies

IRES sequences facilitate cap-independent translation, enabling the expression of multiple genes from a single mRNA transcript [5]. This characteristic renders IRES elements valuable tools for RNA-based therapeutics targeting diverse diseases. In this study, we inserted RNA secondary structures to potentially increase the translation efficacy of the EMCV IRES. Previous reports indicated that RNA secondary structures, such as G-quadruplexes and hairpins, enhance RNA stability and translation efficiency [6,21]. In contrast, we observed that the incorporation of these secondary structures into the EMCV IRES-based vector resulted in decreased translation efficiency. This reduction in protein expression consistently occurred regardless of whether the structures were positioned at the 5' terminus or downstream of the poly(A) tail.

One possible explanation for the discrepancy between our findings and previous reports is that the hairpin structure utilized in our study may have been too small to effectively protect the mRNA terminus. Additionally, the absence of a spacer sequence between the inserted hairpin and the IRES element could have disrupted proper IRES functionality, thereby diminishing translational efficiency.

Since numerous translation-associated factors bind to secondary structures within the IRES, the insertion of hairpins or G-quadruplexes might interfere with factor binding or induce conformational changes in the 3-dimensional architecture of the IRES-based platform. This could potentially hinder ribosomal accessibility or compromise the structural integrity of the IRES, resulting in reduced translation.

### Limitations/suggestions for further studies

In this study, our analysis focused exclusively on the EMCV IRES-based platform because of its previously established excellent translational capabilities. Therefore, further investigations using alternative viral and cellular IRES elements are needed to determine whether inserting these RNA secondary structures similarly reduces translation efficiency across other IRES platforms. Generally, IRES-based platforms exhibit lower translation efficiency compared to cap-dependent systems. Therefore, strategies such as stabilizing the IRES, improving the recruitment of translation-associated factors, or enhancing interactions between the IRES and these factors could enhance IRES translation efficiency. Although RNA structure insertion reduced translation in our current platform, the potential of RNA secondary structures to improve translational efficiency still exists. More precise and strategic design and positioning of RNA structures are required to optimize IRES-based platforms.

### Implications

Enhancement of translational efficiency through RNA structural modifications could facilitate the development of efficient mRNA-based therapeutics, potentially enabling reduced mRNA dosages. This approach may minimize potential side effects and improve cost-effectiveness in therapeutic applications.

### Conclusion

Inserting hairpin or G-quadruplex structures upstream of the 5' UTR or downstream of the poly(A) tail significantly reduced the translation efficiency of EMCV IRES-encoded genes. To effectively enhance translation in IRES platforms, precise 2-dimensional or 3-dimensional structural modeling is required to ensure that inserted RNA structures do not disrupt the native IRES conformation.

## Figures and Tables

**Table 1.** Sequence of structural elements

Abbreviation	Structure	Origin	Sequence (+linker)	Total length (including T7–Not I) (nt)	Position of the structural element
5' UTR modification					
E-G	G-quadruplex	HIV-1	CCAGGGAGGCGTGGCCTGGGCG	1488	25–52
			GGACTGGGGAGTGGCGAG		
E-H	Hairpin	HIV-1	GGTCTCTCTGGTTAGA CCAGAGAGCC	1824	25–55
Poly(A) tail region modification					
E-M1	G-quadruplex	Designed	GGGAAAGGGUUUGGGAAAGGG	1638	1594–1630
E-M2	G-quadruplex + hairpin	Designed	GGGAAAGGGUUUGGGAAAGG	1688	1594–1680
			GGAAGATCAAG**GCTAGC**ACCA		
			UUACCCGCCUUGGGUAAUGG		
			UUGGUAAUGGGUUCCGCCCAU		
			UACCA		
E-M3	G-quadruplex + hairpin	HIV-1	CCAGGGAGGCGTGGCCTGGG	1678	1594–1670
			CGGGACTGGGGAGTGGCGAG		
			**GCTAGC**GGTCTCTCTGGTTAG		
			ACCAGATCTGAGCCTG		
E-M4	G-quadruplex + hairpin	Designed + HIV-1	GGGAAAGGGUUUGGGAAAG	1684	1594–1676
			GG**GCTAGC**ACCAUUACCCGCC		
			UUGGGUAAUGGUGGTCTCTC		
			TGGTTAGACCA GATCTGAGCCTG		

Bold text within the “Sequence (+linker)” column indicates the linker region. nt, nucleotide; UTR, untranslated region; HIV-1, human immunodeficiency virus type 1.

## References

[1] Yang Y, Wang Z (2019). IRES-mediated cap-independent translation, a path leading to hidden proteome. J Mol Cell Biol.

[2] Spriggs KA, Bushell M, Mitchell SA, Willis AE (2005). Internal ribosome entry segment-mediated translation during apoptosis: the role of IRES-trans-acting factors. Cell Death Differ.

[3] Jang SK, Krausslich HG, Nicklin MJ, Duke GM, Palmenberg AC, Wimmer E (1988). A segment of the 5' nontranslated region of encephalomyocarditis virus RNA directs internal entry of ribosomes during in vitro translation. J Virol.

[4] Li Y, Zhang L, Wang L, Li J, Zhao Y, Liu F, Wang Q (2024). Structure and function of type IV IRES in picornaviruses: a systematic review. Front Microbiol.

[5] Ko HL, Park HJ, Kim J, Kim H, Youn H, Nam JH (2019). Development of an RNA expression platform controlled by viral internal ribosome entry sites. J Microbiol Biotechnol.

[6] Lee CY, Joshi M, Wang A, Myong S (2024). 5'UTR G-quadruplex structure enhances translation in size dependent manner. Nat Commun.

[7] Leppek K, Das R, Barna M (2018). Functional 5' UTR mRNA structures in eukaryotic translation regulation and how to find them. Nat Rev Mol Cell Biol.

[8] Chiaruttini C, Guillier M (2020). On the role of mRNA secondary structure in bacterial translation. Wiley Interdiscip Rev RNA.

[9] Spiegel J, Adhikari S, Balasubramanian S (2020). The structure and function of DNA G-quadruplexes. Trends Chem.

[10] Shu H, Zhang R, Xiao K, Yang J, Sun X (2022). G-quadruplex-binding proteins: promising targets for drug design. Biomolecules.

[11] Hansel-Hertsch R, Spiegel J, Marsico G, Tannahill D, Balasubramanian S (2018). Genome-wide mapping of endogenous G-quadruplex DNA structures by chromatin immunoprecipitation and high-throughput sequencing. Nat Protoc.

[12] Lee DS, Ghanem LR, Barash Y (2020). Integrative analysis reveals RNA G-quadruplexes in UTRs are selectively constrained and enriched for functional associations. Nat Commun.

[13] Sissi C, Gatto B, Palumbo M (2011). The evolving world of protein-G-quadruplex recognition: a medicinal chemist’s perspective. Biochimie.

[14] Fracchioni G, Vailati S, Grazioli M, Pirota V (2024). Structural unfolding of G-quadruplexes: from small molecules to antisense strategies. Molecules.

[15] Kiliszek A, Blaszczyk L, Kierzek R, Rypniewski W (2017). Stabilization of RNA hairpins using non-nucleotide linkers and circularization. Nucleic Acids Res.

[16] Bao C, Zhu M, Nykonchuk I, Wakabayashi H, Mathews DH, Ermolenko DN (2022). Specific length and structure rather than high thermodynamic stability enable regulatory mRNA stem-loops to pause translation. Nat Commun.

[17] Solodushko V, Fouty B (2023). Terminal hairpins improve protein expression in IRES-initiated mRNA in the absence of a cap and polyadenylated tail. Gene Ther.

[18] Heinicke LA, Wong CJ, Lary J, Nallagatla SR, Diegelman-Parente A, Zheng X, Cole JL, Bevilacqua PC (2009). RNA dimerization promotes PKR dimerization and activation. J Mol Biol.

[19] Baiersdorfer M, Boros G, Muramatsu H, Mahiny A, Vlatkovic I, Sahin U, Kariko K (2019). A facile method for the removal of dsRNA contaminant from in vitro-transcribed mRNA. Mol Ther Nucleic Acids.

[20] Piekna-Przybylska D, Sullivan MA, Sharma G, Bambara RA (2014). U3 region in the HIV-1 genome adopts a G-quadruplex structure in its RNA and DNA sequence. Biochemistry.

[21] Solodushko V, Kim JH, Fouty B (2025). A capless hairpin-protected mRNA vaccine encoding the full-length Influenza A hemagglutinin protects mice against a lethal Influenza A infection. Gene Ther.

